# The Mediating Role of Social Interactions and Early Psychopathological Symptoms in the Relationship Between Empathy and Prosociality in Young Children with ASD and Neurotypical Peers

**DOI:** 10.1007/s10803-024-06553-6

**Published:** 2024-10-26

**Authors:** Agnieszka Lasota

**Affiliations:** 1 Institute of Psychology, University of the National Education Commission, Krakow, Poland; 2 Jagiellonian University, Krakow, Poland

**Keywords:** Autism spectrum disorder, Typically developing children, Empathy, Prosocial behaviour, Internalising behaviours, Externalising behaviours

## Abstract

This study examined the relationship between empathy, prosocial behaviour, social interactions and early psychopathological symptoms (internalising and externalising behaviours) in children with autism spectrum disorder (ASD) and typically developing (TD) children. A total of 506 parents of children aged 18–48 months participated in this study. The parents of 92 children with ASD and 414 neurotypical children completed the Empathy Questionnaire, the Child Prosocial Behaviour Questionnaire, and the Emotional and Social Development Questionnaire. The results confirmed the direct relationship between empathy and prosocial behaviour in both groups. However, the findings showed a different pattern of the indirect relationship between empathy and prosociality through the social dimensions in the children with ASD compared to their typically developing peers. In the children with ASD, there was only one significant indirect path from empathy to prosocial behaviour – through internalising behaviours (anxiety). Anxiety also played a moderating role in this relationship. The higher the anxiety, the stronger the relationship between empathy and prosociality. In the neurotypical group, social interactions were a significant mediator, strengthening the relationship between empathy and prosocial behaviour. Externalising behaviours weakened this relationship. Intergroup and gender differences were also examined. These findings may have practical implications for social skills training programmes based on behavioural interventions by highlighting the importance of prosocial behaviour for social interaction and protection against psychopathological problems in children with autism and typically developing children.

## Purpose

The development of empathy and prosocial behaviour in childhood has been extensively studied in neurotypical children (Mingins et al., 2021). However, there is relatively little literature on the relationship between empathy and prosociality in children with atypical development, such as children with ASD (Autism Spectrum Disorder). Moreover, many researchers emphasise that empathy deficits in autistic children underlie difficulties in social interactions and psychopathological behaviours (Sari et al., 2021). The aim of this study was to examine the social interactions and early psychopathological symptoms (internalising and externalising behaviours) likely to mediate the relationship between empathy and prosocial behaviour in young children with autism spectrum disorder (ASD) and their neurotypical peers.

### From Empathy to Prosocial Behaviour in Early Childhood

The literature still lacks a clear definition of empathy, but researchers agree that it should be considered as a complex, multidimensional construct (Decety et al., 2016). It can be defined as recognition and understanding of another person’s situation and emotions (Li et al., 2023). It is also the ability to share emotional experiences with others (Wang et al., 2022). Even in early childhood, certain emotional, cognitive and behavioural processes considered to be dimensions of empathy are evident (Li et al., 2023; Rieffe et al., 2010). The first dimensions, already present at birth, is emotional contagion. Infants experience emotional distress in response to another child’s distress (Li et al., 2023; Rieffe et al., 2010) because they have difficulty regulating and controlling their own emotional arousal when confronted with the negative affect of others. Rieffe et al. (2010) state that the next stage of empathy development in childhood includes improved emotional regulation and attentiveness to others’ affective displays. As children grow older, they become sensitive to the emotions of others, and they begin to demonstrate prosocial responses. Prosocial empathy includes intentions to comfort and help, although sometimes the child does not yet show specific behavioural reactions. This dimension of empathy requires not only recognition but also understanding of another person’s emotional state (Li et al., 2023; Rieffe et al., 2010). Empathy is strongly related to prosocial behaviours (Decety et al., 2016; Ryan-Enright et al., 2022; Wang et al., 2022) which also begin in early childhood and change in quantity and quality with age (Eisenberg et al., 2006; Lasota, 2023). Rieffe et al. (2010) found that emotional contagion in young children is linked to higher negative self-regulation, while attention to others’ feelings is a predictor of emotional regulation. Prosocial actions, on the other hand, are a predictor of emotion understanding and prosocial behaviour.

### Empathy and Prosociality in Children with ASD

A growing number of studies has confirmed that empathy develops naturally and effortlessly in most typically developing children, but in autistic children this is a more complex issue (Li et al., 2023). Studies on empathy in children with autism have mostly shown impaired cognitive empathy, but intact affective empathy (Wang et al., 2022), which allows children with ASD to understand the emotions of others and respond accordingly (Deschamps et al., 2014; Wang et al., 2022). However, a growing body of evidence suggests that children with autism have both cognitive and affective empathy disorders (Schnitzler & Fuchs, 2024). Observational studies have suggested that emotional contagion is lower in children with autism than in their non-autistic peers, but parental reports do not support these findings (Deschamps et al., 2014). The study by Li et al. (2023) has shown that autistic children had difficulties in paying attention to others, recognising their emotions, and initiating prosocial activities. Also, fewer prosocial behaviours have often been observed in children with autism compared to TD children in parental reports (Li et al., 2023) and in experimental studies (Wang et al., 2022). However, a meta-analysis by Ryan-Enright et al. (2022) confirmed that the results for autistic children were much more positive about their engagement in prosocial behaviour than diagnostic criteria and some reports suggest.

### Social Interactions in Relations to Empathy and Prosociality

In both cross-sectional and longitudinal studies, empathy and prosocial behaviour have been positively associated with children’s positive social functioning (Eisenberg et al., 2010; Spinrad & Eisenberg, 2017). Cognitive and affective empathy deficits in young children can lead to social and communication problems, such as an increase in maladaptive behaviours or a decline in social understanding (Mingins et al., 2021; Rosen & Lerner, 2016; Sönmez, 2020). It has long been misconceived that individuals with autism lack the ability or desire to establish social relationships (Quinde-Zlibut et al., 2021). Some eye-tracking studies support the hypothesis that children and adults with autism display a reduced attention to social stimuli when compared to typically developing children (Chita-Tegmark, 2016). Researchers have attributed this to reduced social motivation. Social interactions are less satisfying for people with autism, so they orient towards other people less frequently and less spontaneously (Chevallier et al., 2012). As prosocial behaviour is highly sensitive to social context and interpersonal relationships, the socio-cognitive skills such as emotion understanding, perspective taking and ToM enhance social competence and foster empathy and prosociality in childhood (Spinrad & Eisenberg, 2017).

### Exploring the Links Between Empathy, Prosociality and Early Psychopathological Symptoms

Deficits in cognitive, communication and social skills in children with ASD, such as difficulties in empathy (Baron-Cohen, 2010; Li et al., 2023; Quinde-Zlibut et al., 2021), and social adjustment problems (Decety et al., 2016) may contribute to the coexistence of other internalising problems and difficulties, such as anxiety (Mingins et al., 2021; Sönmez, 2020) or externalising problems, such as aggression (Hartley et al., 2008; Rosen & Lerner, 2016; Sari et al., 2021). The relationship between empathy and social anxiety is complex and still unclear (Mingins et al., 2021; van Steensel & Heeman, 2017). Some studies have shown that children with ASD experience higher levels of anxiety than their peers (Mingins et al., 2021). Inconsistent evidence suggests that the level of anxiety in children with autism may be related to their intellectual functioning and thus to cognitive empathy (Tibi-Elhanany & Shamay-Tsoory, 2011). On the other hand, a recent meta-analysis by Pittelkow and colleagues (2021) has found some evidence for a relationship between social anxiety and affective empathy. The direction of the relationship between empathy and anxiety is also ambiguous. Some studies confirm a positive relationship, but in others low levels of cognitive empathy are associated with high levels of social anxiety (Sönmez, 2020).

Another psychopathological factor that may mitigate the relationship between autistic traits and peer relationships is externalisation. Numerous studies using parental and teacher reports have shown that low social competence in peer relationships was strongly associated with externalisation (Sari et al., 2021). Specific features of ASD can lead to maladaptive behaviours (Hartley et al., 2008). Researchers have indicated that rates of aggressive behaviour may be higher in individuals with ASD compared to their neurotypical peers and individuals with other developmental disabilities, although this finding is inconsistent across the literature (Fitzpatrick et al., 2016). Poor language skills, low IQ and maladaptive functioning have been found to be predictors of externalising behaviours in children with ASD (Fitzpatrick et al., 2016; Hartley et al., 2008). The relationship between externalising and internalising behaviours and prosociality among TD children is also complex and ambiguous (Spinrad & Eisenberg, 2017). Many studies of neurotypical children show that empathy is negatively associated with children’s externalising problems, such as aggressive behaviour (Padilla-Walker et al., 2015). Some researchers (Card et al., 2008) have also confirmed a negative relationship between direct aggression and prosocial behaviour in TD children but a positive link between indirect aggression and prosocial behaviour. On the other hand, Wang and Saudino (2015) have found a negative link between prosocial behaviour and internalising problems in neurotypical toddlers.

### Relationship Between Empathy, Prosociality, Internalising, Externalising and Attention Deficit in Children with ASD

Symptoms of attention deficit hyperactivity disorder (ADHD) in early childhood were predictive of later psychiatric symptoms, such as anxiety, depression, aggressive behaviour or conduct problems (Mlodnicka et al., 2024). Moreover, the three core symptoms of ADHD (inattention, hyperactivity, and impulsivity) are often present in children with autism (Montiel-Nava & Peña, 2011). The results of previous studies have shown that children with ASD who also present symptoms of ADHD have greater deficits in social interaction, lower levels of empathy, higher levels of internalising behaviours i.e., anxiety, and lower level of adaptive functioning (Shephard et al., 2019; Dellapiazza et al., 2021). Children with autism and comorbid ADHD have more externalising problems than children with ASD alone, yet fewer than children diagnosed solely with ADHD (Carta et al., 2020). These studies have shown that a diagnosis of ASD alone does not necessarily determine high levels of externalising behaviours in children. As ADHD behaviours such as hyperactivity can be classified as externalising problems (Sari et al., 2021), results from both observational studies and parental reports suggest that children with externalising problems and problems with group socialisation are more likely to be rejected by their peers than children with ASD. On the other hand, higher levels of autistic traits, even in the presence of mild behavioural difficulties, may predict poor peer acceptance (Sari et al., 2021). Yamawaki et al. (2020) also confirmed that children with ADHD and children with comorbid ASD with ADHD had significantly more conduct problems and hyperactivity than children with ASD. On the other hand, the researchers found that children with ASD and comorbid ASD with ADHD had less prosocial behaviour than children with ADHD.

### Gender Differences in Empathy, Prosocial Behaviour and Early Psychopathological Symptoms in Young Children with ASD and TD Peers

The research evidence on gender differences in empathy, prosociality and psychopathological symptoms in young children is inconclusive. While the literature on gender differences in empathy and prosocial behaviour in typically developing children is clearer and differences between girls and boys are more often confirmed (Longobardi et al., 2019), such differences are by no means clear in children with autism. Studies analysing the systemising-empathising profiles of children and adults with ASD have shown that both groups tend to have a hypermasculinised profile, regardless of gender (Harmsen, 2019). The research on gender differences in social interactions has confirmed that neurotypical girls aged 1–4 years outperform boys in socio-emotional development (Bando et al., 2024). Gender-based research among children with ASD has shown that girls with autism (high functioning) report fewer social and communication deficits than boys with autism (Baron-Cohen, 2010; Smith, 2009), as girls are generally expected to be more empathetic or caring. Previous research on neurotypical children has revealed that gender differences in externalising problems seem to begin around age four, while levels of the internalising problems are similar for both genders (Chen, 2010). Preschool TD boys exhibited more externalising problems (including hyperactivity, aggressive behaviour towards younger siblings or other children, frustration, anger) than TD girls (Chen, 2010). However, studies of school-aged children suggest gender differences in both externalising behaviours (more in boys) and internalising behaviours (more in girls) (Levante et al., 2023). Some studies of children with ASD confirm the existence of gender differences in externalising and attention problems (Giarelli et al., 2010). Parent reports and observations suggest that males with ASD have more externalising behaviours and social problems than females (Mandy et al., 2012). Girls with ASD are more likely to have internalising behaviours, e.g. anxiety, depression, than boys with autism (Solomon et al., 2012). On the other hand, Postorino et al. (2015) and Nasca et al. (2020) found no gender differences in internalising and externalising symptoms in preschool and school-aged children with autism.

The existence of gender differences in empathy, prosociality, positive social behaviour and psychopathological symptoms in early childhood should be considered in the light of multiple theories, both neurobiological (structural/functional brain differences) and social modelling, including early socialisation (Rochat, 2023).

### Aim of the Study

The significance of empathy and prosocial behaviour for development of children’s social and emotional competencies makes it essential to understand the relationship thoroughly. This is especially important for children with atypical development, such as ASD. This study examined the relationship between empathy, prosocial behaviour, social interactions, internalising and externalising behaviours and attention deficit as mediators/moderators of this relationship in children with autism spectrum disorder (ASD) and typically developing (TD) children. Internalising and externalising problems are early psychopathological symptoms (Achenbach & Edelbrock, 1978; Blanken et al., 2017) and major axes of childhood behavioural maladjustment (Campbell et al., 2000), whereas social competence contributes significantly to social adjustment (Coplan et al., 2003). In the present study, social competence was operationalised in terms of children’s social interactions (playing with others, inviting and participating in other children’s play). Internalising behaviours were operationalised in terms of anxiety (anxiety about new situations and people, separation anxiety, fearfulness). Externalising behaviours were presented by the children as physical aggression towards another person (e.g. kicking, biting, hitting) or objects (e.g. destroying toys). Attention deficit is understood as difficulties in paying attention, consisting of: distractibility, lack of concentration and difficulties in staying on task.

The following hypotheses were tested:

- H_1_: There will be significant intergroup and gender differences in empathy, prosocial behaviour and positive social interactions between children with ASD and TD children.
- H_2_: There will be significant intergroup and gender differences in early psychopathological symptoms (internalising, externalising behaviours and attention difficulties) between children with ASD and their TD peers.
- H_3_: Empathy will predict prosocial behaviour in children with ASD and TD children.
- H_4_: Social Interactions and early psychopathological symptoms (internalising, externalising behaviours and attention difficulties) will mediate the relationship between empathy and prosocial behaviour in children with ASD and TD children.
- H_5_. Social interactions and early psychopathological symptoms will moderate the relationship between empathy and prosocial behaviour in children with ASD and TD children.

## Method

### Participants and Procedure

A total of 506 parents of children aged between 18 and 48 months took part in the study. Statistical analyses were performed for 92 children with ASD (autism spectrum disorder) and 414 typically developing children. In this study, neurotypical children were matched to children with ASD based on demographic variables. Therefore, children with other health problems, with medical diagnoses, and younger children were excluded from the sample before the analyses. As Polish children under 30 months of age usually do not have a clinical diagnosis, these children were treated as children at high risk for ASD (*N* = 13; 14%). They were in the process of being diagnosed, and they had received psychological opinions indicating symptoms characteristic of autism. The parents of children aged over 30 months provided information about their children’s full diagnosis of ASD (*N* = 79; 86%). All parents gave their written informed consent. Data were collected from parents on a case-by-case basis in diagnostic centres, psychological counselling centres, nurseries, and kindergartens across Poland between 2020 and 2021. An online questionnaire offered via social networks was also used. Information was posted on the author’s website and on popular social networking sites for parents. The study was conducted after approval from the University’s Research Ethics Committee, and all respondents signed an informed consent form.

Information on the demographic variables of the children and their parents is presented in Table 1. The data showed that there were no significant differences between the groups in terms of child’s age or parents’ age. There were also no differences between the groups in terms of parental education (χ² = 0.20, *p* = .98). The controlled variable was also the child’s attendance at a nursery or kindergarten. Among the children, 62% (*N* = 257) of typically developing children and 59% (*N* = 54) of children with ASD attended a nursery or kindergarten. There were no significant differences between the groups (χ² = 5.71, *p* = .13).

**Table 1:** Demographic characteristics of TD children (*N* = 208 girls and 206 boys) and children with ASD (*N* = 38 girls and 54 boys)

		M	Mdn	*SD*	Min	Max	χ²	*p*
Chldren’s age (in months) Girls/Boys	TD	34.3/34.0	35.5/35	8.38/8.20	18	48	0.41	0.938
	ASD	34.1/34.3	34/36	9.00/8.85	18	48		
Maternal age Girls/Boys	TD	31.8/31.8	31/31	4.85/5.13	22/20	46/50	4.47	0.215
	ASD	31.2/33.2	31/32	4.18/4.76	19/24	39/50		
Paternal age Girls/Boys	TD	34.0/34.0	34/33.5	4.99/5.20	24/24	51/52	3.24	0.938
	ASD	33.4/34.9	33/34	6.02/4.78	20/26	56/44		

*Note*: TD: typically developing, ASD: autism spectrum disorder, M: mean, Mdn: median, *SD:* standard deviation, χ²: Kruskal-Wallis’ statistic

### Instruments

#### The Empathy Questionnaire (EmQue)

(Rieffe et al., 2010) was used to assess empathic traits in children from one to five years old. The Polish version (Lasota) consists of 18 items contained in three subscales: Emotional Contagion (EC) (e.g., *When another child is upset, my child needs to be comforted too*), Attention to Others’ Feelings (AOF) (e.g., *My child looks up when another child cries*), and Empathic Prosocial Actions (PRO) (e.g., *When another child gets upset, my child tries to cheer him/her up*). Total scores for all items and subscales were calculated. The Cronbach’s alpha for the total score was 0.81.

#### The Child Prosocial Behaviour Questionnaire (CPBQ)

(Brazzelli et al., 2017) is a parental scale designed to assess prosocial behaviour in young children aged from 12 to 48 months. It consists of 10 items measuring three aspects: Comforting (e.g., *Hugs others when they are upset)*, Sharing (e.g., *Willingly shares toys with other children, when asked*), and Helping (e.g., *Picks up something that I have accidentally dropped and hands it to me*). Only the overall score was used in this study. The Cronbach’s alpha for this scale was 0.84.

#### The Emotional and Social Development Questionnaire (KRES)

(Lasota, 2019) is an 18-item scale designed to assess behavioural and socio-emotional adjustment in young children aged from 8 to 60 months. Positive adjustment includes two subscales: Social Interactions (e.g., My child invites other children to play) and Empathy (e.g., *My child pays attention to the feelings of others*). Maladjustment includes three subscales: Internalising behaviours manifested by Anxiety (e.g., *My child reacts with anxiety to new situations*), Externalising behaviours manifested by Aggression and Difficulties in Regulating Behaviours (e.g., *My child rebels and does not listen to adult’s instructions*), and Attention Difficulties (e.g., *My child is easily distracted during play or tasks*). This instrument has very good accuracy and reliability parameters. In this study, the Cronbach’s alpha for the entire scale was α = 0.79. (0.72 for positive adjustment and 0.80 for maladjustment).

### Statistical Analysis

Statistical analysis was performed using the IBM SPSS software (version 28, PS IMAGO PRO 8.0, Predictive Solutions). Means, standard deviations, and normality tests were performed for all main study variables. The Kruskal-Wallis one-way analysis of variance by ranks with the Bonferroni correction was used to compare the differences between the groups. Hierarchical regression analysis was used to test the prediction of prosociality. Two parallel mediation analyses (Model 4), separately for children with ASD and TD children, and a moderation analysis (Model 1) were estimated using the PROCESS macro for SPSS version 4.2 (Hayes, 2017). Bootstrapped samples were set at 5000 with 95% bias-corrected confidence intervals.

## Results

### Intergroup and Gender Differences in Empathy, Prosocial Behaviour and Early Psychopathological Symptoms 

In order to test the validity of Hypotheses H_1_ and H_2_ an analysis of the differences between the groups was carried out. The data in Table 2 show that there were significant differences in prosociality between neurotypical and children with ASD, both boys and girls. Children with ASD presented lower levels of prosocial behaviour than their neurotypical peers. Girls showed more prosocial behaviour than boys, but only in the TD group. No gender differences with respect to prosociality were found in the group of children with autism. Children with ASD differed significantly from neurotypical children in their levels of empathy (in the total score and two dimensions of empathy: attentions to others’ feelings and empathic prosocial actions). However, in both the ASD and neurotypical groups, there were no intragroup gender differences in this regard. The only exception was prosocial empathy, where differences were found between girls and boys in the TD group. No differences were found between the children in the level of emotional contagion and anxiety. Children with ASD differed from TD children in the level of externalising symptoms and attention deficit, although gender differences were not confirmed in the group of children with autism. Among typically developing children, boys showed higher levels of externalisation than girls. No gender differences were noticed for attention difficulties. Significant differences were found between TD and ASD children, but not gender-based, in their level of positive social interactions.

**Table 2:** Intragroup and intergroup differences between TD children and children with ASD in empathy, prosocial behaviour and early psychopathological symptoms

	TD children		ASD children					
	Girls (1)	Boys (2)	Girls (3)	Boys (4)				
	Mrang	Mrang	Mrang	Mrang	χ²	p	ε²	Post-hoc test
Prosocial	309.3	254.9	158.6	100.1	106.07	< .001	0.210	2 < 1, 4 < 1, 3 < 1,3 < 2,4 < 2
Empathy	292.0	261.8	164.5	135.9	64.24	< .001	0.127	4 < 1, 3 < 1,3 < 2,4 < 2
EC	264.7	262.2	202.8	212.5	10.91	0.012	0.021	-
PRO	300.7	252.8	131.2	172.6	71.94	< .001	0.142	2 < 1, 4 < 1, 3 < 1,3 < 2,4 < 2
AOF	275.7	262.1	202.9	171.1	27.74	< .001	0.054	4 < 1, 3 < 1, 4 < 2
Emp	310.1	246.1	170.3	122.1	88.87	< .001	0.175	2 < 1, 4 < 1, 3 < 1,3 < 2,4 < 2
Internal.	252.5	247.1	267.2	272.0	1.62	0.655	0.003	-
External.	211.2	251.7	341.5	361.2	62.00	< .001	0.121	2 < 1, 4 < 1, 3 < 1,3 < 2,4 < 2
Att Def.	232.1	240.5	314.3	342.4	33.02	< .001	0.065	4 < 1, 3 < 1,3 < 2,4 < 2
Soc. Int.	281.5	259.5	194.7	164.3	35.45	< .001	0.070	4 < 1, 3 < 1, 4 < 2

*Note*: Mrang: mean range; χ²: Kruskall-Wallis test result; p: p-value; ε2: effect size; In post-hoc test 1: typically developing girls, 2: typically developing boys, 3: girls with ASD, 4: boys with ASD; EC: empathy emotional contagion, PRO: empathy prosocial actions, AOF: empathy attention to others’ feelings, Emp: empathy KRES, Internal: internalising behaviours, External: externalising behaviours, Att. Def.: attention deficit, Soc. Int.: social interactions

### Empathy as a Predictor of Prosociality in Children with ASD and TD Children

To assess whether empathy is a predictor of prosocial behaviour in children with ASD and their TD peers (Hypothesis 3), a stepwise hierarchical regression analysis was conducted, with a test probability of *p* ≤ .05 as the inclusion criterion and *p* ≥ .10 as the exclusion criterion. The model included the following predictors: empathy dimensions (EC, AOF and PRO) in the first step, positive social relations, and internalising and externalising behaviours in the second step, and gender in the third step. The results showed that significant predictors of prosocial behaviours in typically developing children were prosocial empathy (standardised β = 0.36, t = 8.50, *p* < .001), social interactions (β = 0.25, t = 6.11, *p* < .01), externalising behaviours (β = − 0.25, t = -5.97, *p* < .001) and female gender (β = 0.09, t = 2.22, *p* < .05). These variables accounted for ΔR^2^ = 0.36. The predictors of prosocial behaviour in children with ASD were prosocial empathy (β = 0.52, t = 6.34, *p* < .001), positive social interactions (β = 0.22, t = 2.69, *p* < .01), anxiety (β = 0.19, t = 2.30, *p* < .05) and attention difficulties (β = − 0.25, t = -2.97, *p* < .01). These variables accounted for ΔR^2^ = 0.41.

### Social Interactions and Early Psychopathological Symptoms as Mediators Between Empathy and Prosocial Behaviour

**Fig. 1:**
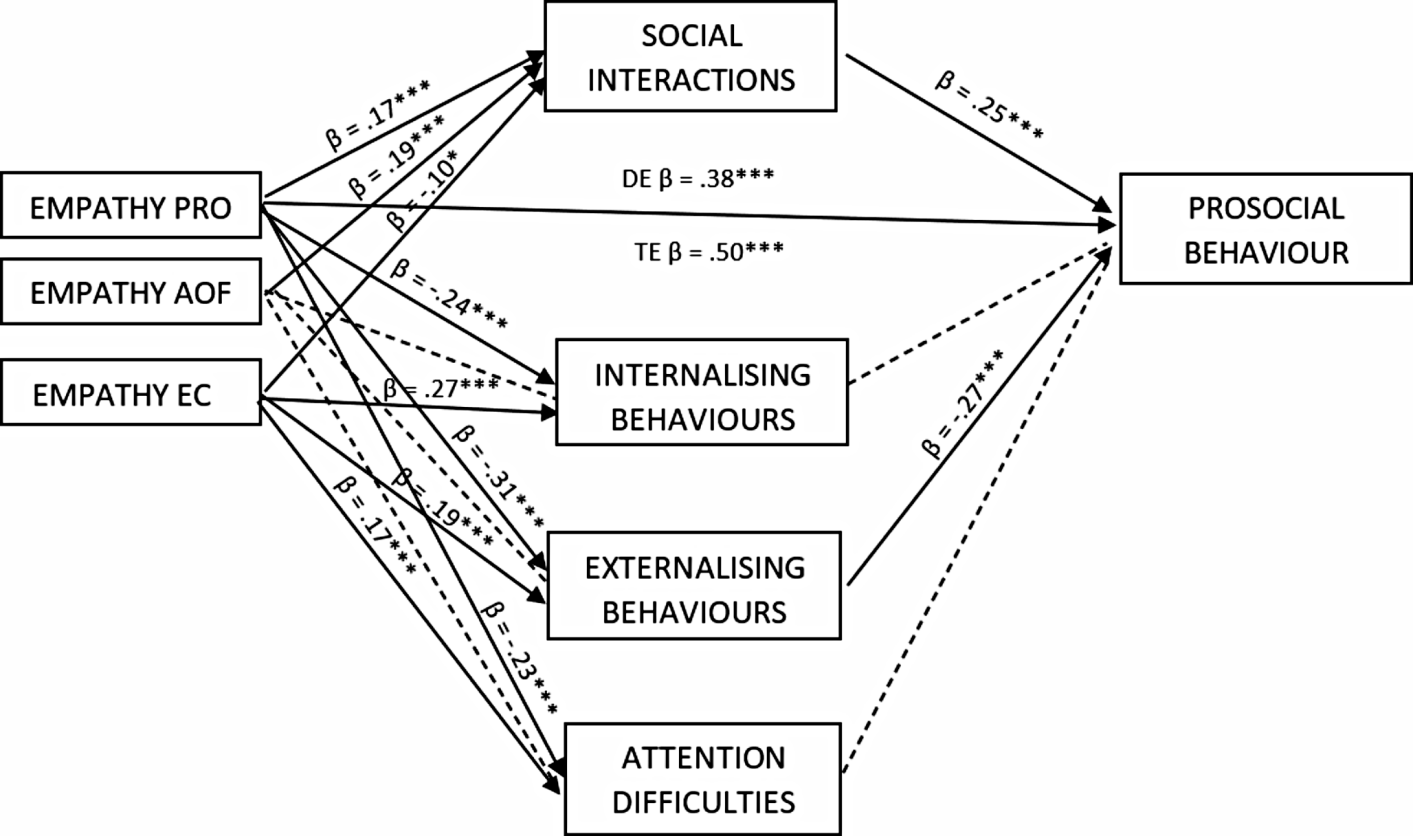
Parallel mediation model of empathy on prosocial behaviour in TD children using social interactions and early psychopathological symptoms as mediators. Note: Empathy PRO: prosocial actions, Empathy AOF: attention to others’ feelings, Empathy EC: emotional contagion. Standardised coefficients are presented. * *p* < .05, ** *p* < .01, *** *p* < .001; DE: direct effect, TE: total effect

A multiple mediation model was used (model 4) to examine whether positive social interactions, early psychopathological symptoms and attention difficulties mediated the association between empathy (AOF and EC as covariates) and prosocial behaviour (Hypothesis 4). All outcomes were standardised. In typically developing children (Fig. 1), the empathy dimension–prosocial action, was positively related to the outcome variable and all mediators. It showed a positive correlation with social relations, and an inverse correlation with three negative behaviours (internalising behaviours, externalising behaviours, and attention deficit). Attention to others’ feelings was only related to social interactions. Emotional contagion was negatively related to positive socialisation and positively correlated with all negative dimensions. Social relationships (positive) and externalising behaviours (negative) were significantly related to prosocial behaviour. The indirect effect of empathy on prosocial behaviour through social interactions in TD children was found to be significant (β = 0.043, SE = 0.015, CI [0.02, 0.07]). The indirect effect of empathy on prosocial behaviour through externalising behaviours was also significant (β = 0.085, SE = 0.018, CI [0.05, 0.12]). The indirect effects accounted for 24%. This model was a good fit to the data (direct ΔR^2^ = 0.25, *F*(3,410) = 44.8, *p* < .001, total ΔR^2^ = 0.36, *F*(7,406) = 32.73, *p* = .001).

**Fig. 2:**
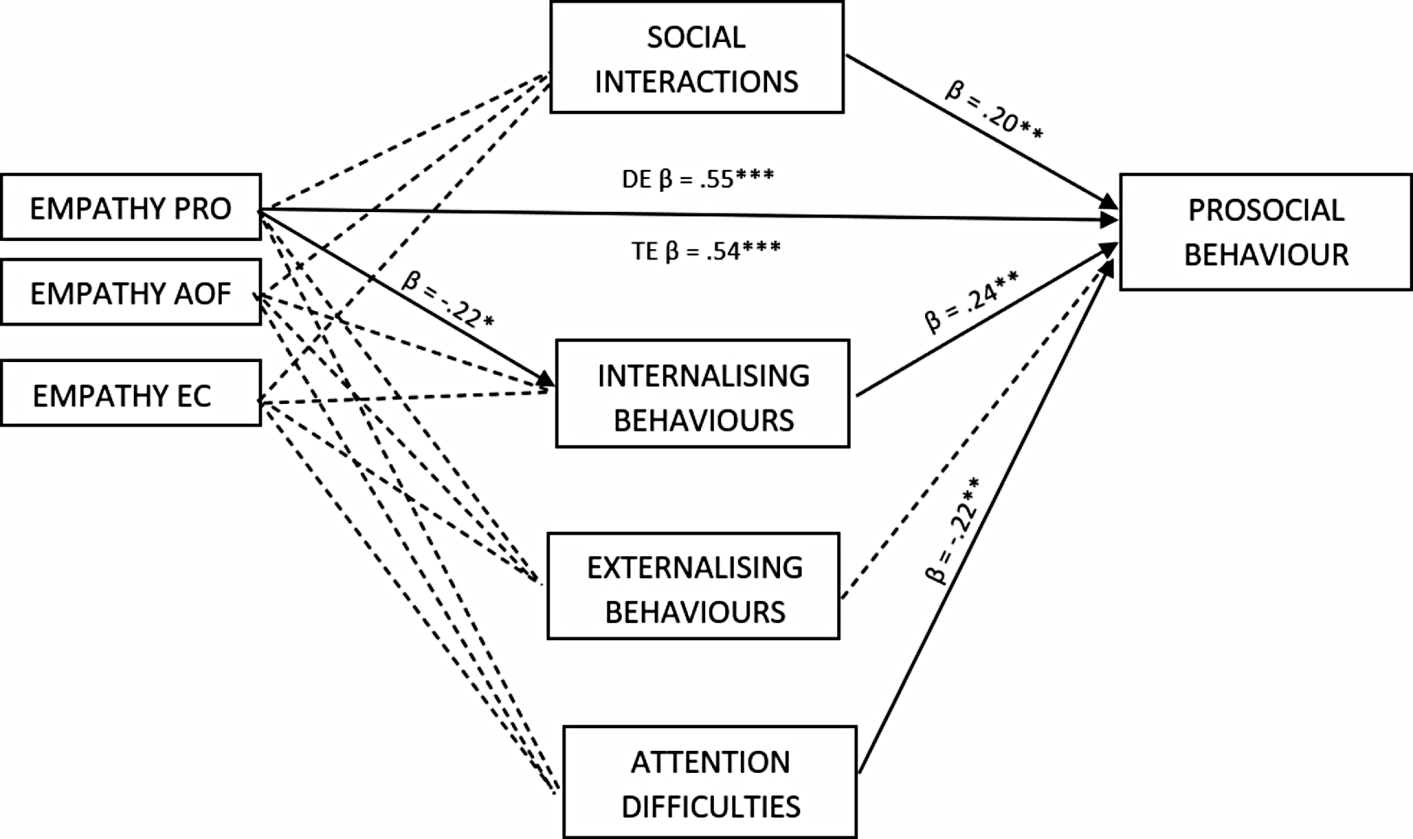
Parallel mediation model of empathy on prosocial behaviour in children with ASD using social interactions and early psychopathological symptoms as mediators. Note: Empathy PRO: prosocial actions, Empathy AOF: attention to others’ feelings, Empathy EC: emotional contagion. Standardised coefficients are presented. * *p* < .05, ** *p* < .01, *** *p* < .001; DE: direct effect, TE: total effect

In children with autism spectrum disorder (Fig. 2), none of the dimensions of empathy were directly related to positive socialisation. No direct relationship was found between empathy and the mediators: attention deficit or externalising behaviours. The prosocial dimension of empathy was significantly negatively associated with internalising behaviours. This type of empathy was also directly related to prosocial behaviour. Social interactions and anxiety were positively correlated with prosocial behaviour, while attention deficit was negatively associated with prosociality in children with ASD. Only one indirect path from empathy to prosocial behaviour was found to be significant – through internalising behaviours (IE β = − 0.053, SE = 0.035, CI [-0.13, − 0.001]). Prosocial behaviours were directly positively correlated with social interactions and anxiety, and negatively correlated with attention difficulties. This model was a good fit to the data (direct ΔR^2^ = 0.31, *F*(3,88) = 12.94, *p* < .001, total ΔR^2^ = 0.45, *F*(7,84) = 9.81, *p* = .001).

### Social Interactions and Early Psychopathological Symptoms as Moderators of the Relationship Between Empathy and Prosocial Behaviour

**Fig. 3:**
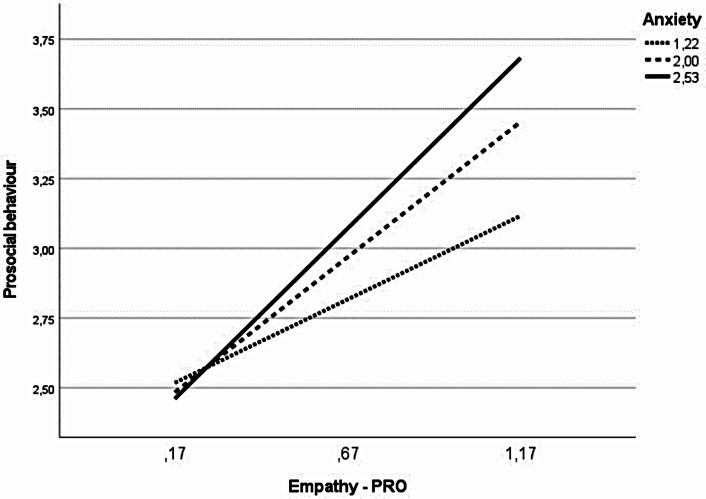
A visual representation of the moderating effect of empathy-pro on prosocial behaviour at low, average, and high levels of internalising behaviour (anxiety) in children with ASD

Additionally, to check whether social interactions, externalisation, internalisation or attention difficulties moderates the relationship between empathy and prosociality in children with ASD and TD children (Hypothesis 5), moderated analyses were carried out. Given the exploratory nature of this hypothesis, analyses were conducted with three dimensions of empathy (EC, AOF, PRO) as independent variables and prosocial behaviour (total score) as the dependent variable. The positive and negative dimensions of social functioning (social interactions, internalising, externalising and attention difficulties) were tested as moderators of this relationship. All possible models were tested separately on a group of typically developing children and a group of children with autism. Only internalising behaviour (anxiety) was found to be a moderator of the relationship between empathy and prosocial behaviour in children with ASD (Fig. 3). The analysis confirmed that the model was a good fit to the data (*F*(3,88) = 15.32, *p* = .001, ΔR^2^ = 0.35). Inclusion of the interactive component in the model (*F*(1,88) = 4.79 *p* = .05) significantly increased the percentage of explained variation of the dependent variable by 3.6%. The strongest effect was found for the highest level of anxiety (non-standardised b = 1.26, se = 0.21, t = 5.95, *p* = .001, CI [0.84, 1.68]), followed by moderate anxiety (b = 0.94, se = 0.14, t = 6.52, *p* = .001, CI [0.65, 1.23]). The weakest effect was found for low anxiety in children with autism (b = 0.62, se = 0.20, t = 3.07, *p* = .003, CI [0.22, 1.02]).

## Discussion

The study aimed to investigate the relationship between empathy and prosocial behaviour in young children with ASD and typically developing children. It also looked at how other social factors might mediate or moderate the relationship between the study variables.

The study found that children with ASD were less empathetic than their TD peers, more specifically in terms of overall empathy and its two dimensions: attention to others’ feelings and prosocial empathy. No differences were found in emotional contagion. This is consistent with previous research findings based on parental reports (Li et al., 2023). The results show that children with autism have difficulty paying attention to other people’s emotions and engaging in prosocial actions towards another person. When exposed to someone’s emotional expressions, autistic children may feel stressed because they have difficulty understanding the emotions being conveyed. To cope with this stress, individuals with ASD may shift their focus elsewhere (Markram & Markram, 2010; Li et al., 2023).

Children with ASD were assessed by their parents as less prosocial than TD children, which is in line with the previous findings (Li et al., 2023). Prosocial behaviour requires three social-cognitive abilities: recognising the negative experiences of others, knowing how to respond, and being motivated to act (Ryan-Enright et al., 2022). Therefore, children with ASD may have more difficulty understanding and predicting the emotions or behaviours of others, and they may be less motivated to act prosocially. Moreover, prosocial behaviour have been defined as comforting, helping, and sharing behaviours. Children with autism may express their prosocial behaviour differently. Instead of comforting, they may show care and support by quietly and attentively listening to others (Crompton et al., 2020).

Children with autism spectrum disorder engaged in social interactions significantly less frequently compared to their TD peers. On the other hand, children with ASD had significantly higher levels of externalising symptoms and attention difficulties compared to their TD peers. This is consistent with the findings of other researchers (Mingins et al., 2021; Tsou et al., 2021) suggesting that children with ASD stand an increased risk of challenging behaviours and decreased adaptive behaviours.

The present study has found that levels of internalising symptoms, i.e., levels of anxiety, did not differ between the two groups of children. These findings are contrary to other studies (Mingins et al., 2021; van Steensel & Heeman, 2017), that examined older children with ASD. The reason for this may be that the study involved parents evaluating very young children (aged 1.5-4 years) experiencing separation anxiety, which caused both groups of parents to observe anxiety in their children. It is possible that these differences may increase with age (anxiety decreases in TD children, while it increases in children with ASD), as this was confirmed in the study of older children with ASD (Mingins et al., 2021).

The results of this study confirmed gender differences only in the group of typically developing children: the girls had higher levels of prosocial empathy and behaviour, while the boys had a higher level of externalising symptoms. This is consistent with previous research showing that girls and women often outperform boys and men on standardised tests measuring empathy, social sensitivity and emotion recognition (Mingins et al., 2021). No gender differences were observed in the group of children with ASD, which is in contrast to previous findings (Baron-Cohen, 2010; McChesney & Toseeb, 2018). The explanation is perhaps to be found in the age of the children studied and their attendance of a nursery or kindergarten, where all children undergo the same process of socialisation regardless of gender. Although many studies confirm that TD boys and boys with ASD face lower social pressures and expectations regarding empathic and prosocial behaviour compared to girls (Smith, 2009), these findings have shown that such gendered social expectations and stereotyped social roles are not yet transparent in young children, especially those attending a nursery or kindergarten (Lasota, 2023). Perhaps the presence of siblings (Ben-Itzchak et al., 2016) and natural contact with other children in the family or at a nursery contributes to better functioning in young children with ASD, irrespective of their gender. This is consistent with the social learning theory, which posits that prosocial behaviour develops when (neurotypical) children learn social norms of reciprocity and social responsibility (Ryan-Enright et al., 2022).

The results confirmed that empathy was directly related to prosocial behaviour in children with autism as well as in their neurotypical peers. However, only prosocial empathy was a significant predictor of prosocial behaviour. The other two dimensions of empathy, emotional contagion, and attention to others’ feelings, were not directly related to the level of prosocial behaviour observed by the parents. This finding should not be surprising, as prosocial empathy involves emotions that lead a person to engage in helping or comforting behaviours. Experiencing prosocial empathy requires understanding others’ emotions and social roles. As children begin to recognise the basic emotions, they simultaneously develop prosocial behavioural intentions (Tsou et al., 2021).

The finding revealed that social interactions correlated with empathy and prosocial behaviour only in typically developing children. Attention to others’ feelings and prosocial empathy were positively associated, while emotional contagion was negatively associated with social relationships in TD children. Social interactions were a significant mediator that strengthened the relationship between empathy and prosocial behaviour in neurotypical children. However, in children with autism spectrum disorder, the relationship between empathy and prosociality was not mediated by positive social interactions. This result can be explained by the fact that impaired social attention ultimately deprives the child of relevant social learning experiences, causing an imbalance in the reception of social and non-social stimuli, and subsequently disrupting their social and cognitive development (Wang et al., 2022).

These findings confirmed that early psychopathological symptoms mediate the relationship between empathy and prosociality in young children. In typically developing children, two out of three dimensions of empathy were found to be associated with psychopathological symptoms. Emotional contagion was positively and prosocial empathy negatively associated with all negative socialisation behaviours. This means that typically developing children who have difficulty regulating their own emotional arousal and understanding other people’s emotions are more likely to experience externalising symptoms. Moreover, externalising behaviour emerged as a significant mediator weakening the relationship between empathy and prosocial behaviour.

In children with autism spectrum disorders, a completely different relationship was found. Interestingly, and in contrast to previous findings in older children with ASD (Fitzpatrick et al., 2016; Montiel-Nava & Peña, 2011), empathy was not correlated with externalising behaviour or attention difficulties in this study. Social interaction was a positive and attention difficulties a negative predictor of prosocial behaviour in children with ASD. The mediator of the relationship between empathy and prosocial behaviour was found to be internalising behaviour. The negative association between empathy and anxiety was in line with previous research (Sönmez, 2020), but in contrast to others (Gambin & Sharp, 2018). On the other hand, these findings show that children with ASD who felt anxious exhibited more prosocial behaviour.

Furthermore, anxiety was found to act not only as a mediator, but also as a moderator in the relationship between empathy and prosocial behaviour in children with ASD. This relationship between empathy and prosociality was the strongest at higher level of anxiety. Perhaps anxiety is a motivator for children with ASD to observe and imitate the behaviour of other children. Tibi-Elhanany and Shamay-Tsoory (2011) have found that autistic individuals with high social anxiety showed greater sensitivity and attentiveness to other people’s states of mind. High levels of anxiety may make children with autism more attentive to others’ emotions. Children with ASD who have higher levels of anxiety may also have better mentalising and empathising skills, leading to more prosocial behaviour. The findings are also consistent with those of Rosen and Lerner (2016), who confirmed that in adolescents with ASD, improvement in facial emotion recognition was enhanced by internalising symptoms but attenuated by externalising symptoms. The results of this study confirm that the relationship between empathy, prosocial behaviour and anxiety in children with ASD is not clear-cut and is much more complex.

### Limitations

This study has some potential limitations that should be considered. First, it was a cross-sectional study based solely on parental reports. The results should be interpreted cautiously because empathy traits and prosocial behaviour in daily life were parent-reported, indicating the potential measurement error. Second, 14% of the children in the ASD group were under 30 months old and had not yet been clinically diagnosed. Third, the researchers did not have access to a full diagnosis of the children with autism spectrum disorders, which limited the inclusion of other variables in the analyses, such as intelligence or communication levels of the children with ASD and TD group. It is also likely that there were differences in general developmental and language skills between the groups, making it difficult to conclude how much of the findings are specific to autism and how much is due to broader developmental delays. Moreover, this study considered prosocial behaviour as a total variable. Including of three dimensions of prosocial behaviour may have revealed additional interesting relationships. Finally, a limitation of the study is that the sample consisted only of Polish parents and therefore the results cannot be generalised.

Despite these limitations, these findings represent one of few studies that have examined the relationship between empathy and prosocial behaviour while additionally considering social interactions and early psychopathological symptoms in children with ASD compared to their TD peers. This study found that in early childhood, the relationship between empathy and prosocial behaviour differs between children with ASD and neurotypical children, based on their behavioural and social-emotional functioning. This study may have practical implications for social skills training programmes based on behavioural interventions. The findings suggest that intervention programmes to develop emotional-social skills should target a specific group of children in early childhood. To strengthen the link between empathy and prosociality, the focus should be on reducing externalising behaviours in typically developing children, and on reducing internalising behaviours in children with ASD.

## Conclusions

These findings add to the literature on the difficulties experienced by children with ASD from an early age in recognising emotions, caring for others, and responding prosocially. The results of this study also show a different pattern of the relationship between empathy and prosociality, including positive and negative social factors as mediators, in children with ASD compared to TD children. This multifaceted approach to understanding the relationship between empathy, prosociality and early psychopathological symptoms may help to reduce the negative stereotypical image of the behavioural and socio-emotional problems in children with autism. It may also facilitate understanding of the social and emotional challenges faced by children with autism in the process of developing multidimensional empathy and prosociality (Li et al., 2023).
